# When the cure leaks: corruption, health spending, and health outcomes in Malawi

**DOI:** 10.3389/fpubh.2025.1715969

**Published:** 2026-01-12

**Authors:** Emmanuel George Yusufu, Bertha Chipo Bangara, Lloyd George Banda

**Affiliations:** 1 Department of Economics, School of Law, Economics and Government, University of Malawi, Zomba, Malawi; 2 Institute for Economics, Freiburg University, reiburg im Breisgau, Baden-Württemberg, Germany; 3 Department of Political Science, Stellenbosch University, Stellenbosch, Western Cape, South Africa

**Keywords:** corruption, health expenditure, health outcomes, ARDL, Malawi

## Abstract

**Introduction:**

The common assumption that higher investment leads to improved development outcomes is challenged in the health sector by widespread corruption practices—a paradox that fundamentally challenges the effectiveness of public health spending. The current paper sought to examine the impact of health spending on health outcomes and how this relationship changes under the auspices of increasing corruption trends in Malawi.

**Methods:**

The study employed the autoregressive distributed lag approach, bounds cointegration, and the error correction model to establish the long-run relationship among the variables for annual time series data from 1990 to 2023.

**Results:**

Empirical findings revealed that a one-unit increase in corruption reduces life expectancy by 0.146 percentage points and increases infant mortality by 0.0226 percentage points in the long run. In addition, while the long-run coefficient of health spending increases life expectancy and reduces under-five mortality, the contingent effect of corruption worsens the results by 0.317 and 0.149 percentage points, respectively.

**Conclusion:**

These results concur with our location of corruption within the institutional hypothesis—which posit that the quality and structure of institutions determine economic performance, and the Solow swan growth model in which we see corruption as an externality and source of inefficiencies, reducing the productivity of public investment in health (K) and the rate of technological progress (A(t)). Importantly, we made various policy suggestions, such as tying health expenditure to measurable outcomes to ensure efficiency and reduce leakages of public funds.

## 1 Introduction

The health sector remains a cornerstone of socio-economic development, due to its multiplier effects on other economic sectors through human capital accumulation and productivity (1, 2). Yet, despite the intuitive expectation that greater public spending should translate into better population health, empirical studies show that health outcomes do not always improve proportionally with expenditure, particularly in low- and middle-income countries (LMICs) where governance failures are prevalent (3, 4). Globally, corruption accounts for approximately 7.3% of total health spending, exceeding the annual investment required to achieve universal health coverage (4), which underscore concerns that institutional leakages can nullify the benefits of budget increases. The trends are also observed in Europe, Asia and Sub-Saharan Africa, where bribery, procurement fraud and embezzlement humper service delivery and weaken health systems (5–8).

While African governments have committed to increasing health investment by subscribing to the Abuja Declaration (2001) and the Malabo Declaration (2014), most countries continue to underperform on expenditure targets and health indicators (9–12). Malawi exemplifies this paradox. Despite repeated policy commitments to universal health coverage and substantial donor support, health outcomes remain among the lowest globally, with persistent maternal and infant mortality, disease burden, and widening healthcare inequalities (13–17). Corruption in procurement, fund management and service delivery remains systemic, with up to 30% of health resources lost annually (15, 16, 18–22). These inefficiencies raise a critical question: Does higher health spending yield improved outcomes in Malawi when corruption persists?

Existing work has largely examined either the direct effect of health expenditure on outcomes (23–26) or the independent impact of corruption on population health (27–32). Within Malawi, Mhango and Chirwa (33) assessed governance effects on infant mortality, but did not explore (i) life expectancy as a broader outcome measure, (ii) corruption as a moderating factor on the spending–outcome nexus, or (iii) long-run dynamic relationships using extended time-series data. Thus, no study to date evaluates how corruption conditions the effectiveness of health expenditure on life expectancy in Malawi over time, leaving a substantive empirical gap.

To address this gap, the current study employs the Autoregressive Distributed Lag (ARDL) econometric approach to analyse annual time-series data from 1990 to 2023. The ARDL approach is appropriate because the health outcome variables and its determinants used in this study exhibit mixed integration orders (I(0)/I(1)), and the technique performs reliably with small samples while allowing estimation of both short- and long-run relationships (34–36). This design enables us to interrogate whether improvements in public spending translate into better health outcomes while accounting for the contingent effects of corruption.

Accordingly, this study examines:

(1) the effect of health expenditure on life expectancy in Malawi, and
(2) the moderating influence of corruption on that relationship.

By situating the analysis within institutional theory and the Solow-Swan growth framework, the study show how corruption functions as an efficiency-reducing externality that weakens the productivity of health investment. Findings offer policy-relevant insights for Malawi’s pursuit of Vision 2063 and universal health coverage reforms.

## 2 Related literature

### 2.1 Facts about the healthcare system and Corruption in Malawi

While Malawi commendably achieved Millennium Development Goal (MDG) 4 (33), the country suffers a high disease burden, including tuberculosis, malaria, and HIV (14). According to the Human Development Index, life expectancy in Malawi was 65.18 years as of 2022. Malawi would be better off regarding health outcomes without persistent health financing gaps. For example, in 2013, government expenditure was US$177 million and an additional US$207 million from donors, yet the required amount was US$535 million (14). While Malawi has transhistorical problems financing its health system with a persistent deficit, the fiscal year 2024/25 marked a significant increase in health budget allocation (37). That is, the sector received an allocation of MK723 billion, an increase from MK330 billion allocated in the 2023/24 fiscal year, representing an increase of 119.1 percent. While desirable, the increase is overwhelming, and caution about its sustainability should be exercised, as it may be politically motivated ahead of the general elections in 2025. Henceforth, one of the motivations of this study is to examine whether such an increase in public health spending increases health outcomes contingent on persistent corruption.

Despite persistent financing gaps, the health sector is the breeding ground for corruption in the country. For instance, during the time of crisis when people need healthcare assistance more than ever, officials had the tenacity to swindle COVID-19 allocated funds into their own pockets, with public officials and private sector officials colluding to misspend about US$1.3 million pandemic funds through inflated procurement expenses (18). This malpractice occurred despite the country having a US$194 million financial deficit to successfully implement its COVID-19 preparedness and response plan of US$213 million (19). Evidence from Afrobarometer surveys confirm that Corruption in Malawi is institutionalised (20, 21). A study using a purposive sampling of 22 representatives from various public and private, local and international organisations established that there is a lack of accountability in health-funded programmes and that political, structural, and financial factors challenge the overall governance in the national health system (22).

### 2.2 Theoretical review

The theoretical foundation for understanding the contingent role of corruption on the nexus between health expenditure and health outcomes draws upon the institutional hypothesis. Despite many notable economists contributing to this theory, the standard definition is that institutions are the rules, norms, and frameworks that shape human interaction, including legal systems, property rights, governance structures and social norms (38, 39). It suggests that the fundamental determinants of economic performance and development are the quality and structure of its institutions (39, 40). Corruption benchmarks as a breakdown in institutional accountability, transparency, and governance. According to the institutional hypothesis, public spending potential is limited by poor governance structures (41). This phenomenon can be attributed to the diversion of resources from healthcare to personal enrichment or political patronage, overpricing or low-quality supplies in procurement due to bribery. This, in turn, reduces the expenditure values and discourages donor funding or international support.

Another premise of institutional hypothesis is that weak institutions create environments where public goods, like healthcare, fail to reach those in need (42). Resultantly, this leads to inequalities in health care access, which in turn affects the vulnerable population, hence worsening health outcomes. Sibanda et al. (43) noted that the root cause of low life expectancy rates is deeply embedded in weak institutional frameworks. Improving accountability within the health sector can ensure that health budgets are effectively translated into better health outcomes. Strong institutions play a vital role in this process by providing the necessary structure and governance to support the efficient use of resources and the delivery of high-quality healthcare services (44).

### 2.3 Empirical review

#### 2.3.1 Government health expenditure and health outcomes

A study by Borghi et al. (23) on health financing at the district level in Malawi for 2006–2011 used Pairwise Correlation, Concentration Indices and Lorenz Curves for data analysis. The study established that districts with higher out-of-pocket payment levels had higher 1–59-month mortality rates, while districts with higher levels of domestic and external finance had lower mortality rates. This call suggests that increased funding likely improves health care access and quality, leading to better survival rates of children. Onofrei et al. (24) assessed the relationship between public health spending and outcomes among EU and developing countries. The study results showed that public health expenditure and health outcomes are in a long-run equilibrium relationship, and the status of health expenditure can improve life expenditure and reduce infant mortality. Bokhari et al. (25) examined the relationship between a country’s per capita government expenditure and per capita income on under-five mortality and maternal mortality. They used the Instrumental Variable Technique (GMM-H2SL) and established that government health expenditure is essential in improving health outcomes.

Bokhari et al. (25) focused on under-five and maternal mortality in all developing countries and established that health expenditure and economic growth are important contributors. Furthermore, Kamanda et al. (26) explored the interaction of health expenditure, health outcomes and economic growth in sub-Saharan Africa using vector autoregressive models and Granger causality. This study established that health expenditure and health outcomes positively impact economic growth, suggesting that policies promoting health spending can support economic progress.

#### 2.3.2 Corruption and health outcomes

A parallel body of work examines corruption as a determinant of poor health outcomes. Achim et al. (27) and Li et al. (28), using multi-country panels, show that corruption increases mortality and reduces life expectancy through reduced service quality, informal payments, and resource diversion. Naher et al. (45) studied the influence of corruption and governance on delivering health care services in low and middle-income countries of South and Southeast Asia. They conducted a scoping review following the Preferred Reporting Items for Systematic Reviews and Meta-Analysis (PRISMA), and the data were analysed using the mixed studies review method. The study established that corruption hinders the delivery of quality healthcare services, leading to poor health outcomes. Toffolutti et al. (29) studied 135 regions in 17 Sub-Saharan countries on the association of bribery and maternal mortality. Using a linear probability model, the study established that a 10 percent increase in the prevalence of bribery is associated with 41 additional deaths for every 1,000 pregnancy deaths. Sharma (30) examined the impact of economic freedom on four indicators of health: life expectancy, infant mortality, under-five mortality rate and neonatal mortality rate. The study used a panel data set of 34 sub–Saharan African countries from 2005 to 2016, and used fixed effects regression, and two-stage least squares regression (2SLS). The study results showed that higher levels of economic freedom reduce mortality rates and increase life expectancy.

Lio and Lee (31) conducted a cross-country study on the impact of corruption control on health outcomes. This study used cross-country panel data covering 119 countries from 2005 to 2011, and employed two-way fixed effects and the two-stage least squares approach. The study established that corruption is negatively associated with a country’s health outcomes, and better control of corruption can lead to longer life expectancy and lower infant and under-five mortality rates. Nichter et al. (32) empirically established that anti-corruption audits significantly decreased early-life mortality in Brazil. The study suggested that governments can potentially improve health outcomes through anti-corruption intervention. Only Mhango and Chirwa (33) assessed the links between public health spending, governance and health outcomes in Malawi. Their study showed that governance matters for infant mortality, yet they do not evaluate life expectancy, nor model corruption as a moderator of expenditure effectiveness.

Drawing from both theoretical and empirical insights, the interaction of health expenditure and health outcomes can hardly be assumed to operate uniformly across institutional environments. In contexts like Malawi, where up to 30% of funds are lost to leakages and procurement abuses (15, 16, 23–27), increases in government spending may produce weaker or even negligible health gains when corruption persists. Correspondingly, the present study models corruption as a moderating channel influencing how effectively health expenditure translates into life expectancy improvement.

## 3 Methods and data

### 3.1 The outcome variable

The study used life expectancy (LEXP) as an outcome variable. Life expectancy represents the average number of years a newborn is expected to live if current mortality patterns remain constant throughout their lifetime (4). We followed the literature insights (24, 46–48) and we used two public health outcomes indicators, namely life expectancy at birth and infant mortality. Data for LEXP were obtained from the World Development Indicators (WDI). The under-five mortality rate variable was used to replace LEXP in our regression only for robustness checks of our findings.

### 3.2 Explanatory variables

Health expenditure is the primary explanatory variable of interest. We used the indicator current health expenditure per capita (HXPC) from WDI. The indicator measures all healthcare goods and services consumed annually. It reflects the financial resources allocated to health per person in a country and combines government funding (30%) with substantial private spending (40% out-of-pocket), exposing households to catastrophic costs (4). As used in most studies, we expect a positive impact of health expenditure on life expectancy in Malawi (46, 49). Including this variable as an explanatory variable is important because it captures total health expenditure, including donor and private contributions, which are significant in Malawi’s mixed financing system.

Realising that health budget allocation has barely resulted in improvement in health outcomes, we employ the corruption indicator named Control of Corruption (CRC) from World Governance Indicators (WGI) as a contingent variable in the health expenditure-outcomes nexus. CRC reflects expert assessment and the perception of the extent to which public power is exercised for private gain, including both petty and grand forms of corruption, as well as capture of the state by elites and private interests (50). While widely applied in governance–development studies due to long time coverage and comparability across countries, perception-based measures are susceptible to reporting bias, measurement error, and sensitivity to political events. These limitations are acknowledged, but the index remains the most consistent long-run corruption proxy available for Malawi and is therefore appropriate for time-series modelling. Unsurprisingly, the CRC has been used in many studies examining the impact of corruption on health outcomes (5, 51).

### 3.3 Control variables

In this study, we recognise that there are several determinants of health outcomes that we cannot specify in a short time series in a country-specific study. Therefore, we control for national output and education, which have been popularly used in similar studies (33, 43). National output is measured by gross domestic product per capita (GDPPC), which measures the total value of all final goods and services produced within a country in a given year, divided by midyear population (52). On the other hand, education is measured by mean years of schooling (MYS), which captures the average number of completed years of formal education received by persons aged 25 and older, excluding repeating grades (53). Although demographic and service-delivery variables such as immunisation coverage, HIV prevalence, and maternal health indicators influence life expectancy, they were not included in our model mainly because of high collinearity with health expenditure and income and the study’s macro-structural emphasis. In addition, immunisation coverage and HIV prevalence have limited historical coverage in Malawi.

### 3.4 Empirical analysis

#### 3.4.1 Empirical framework

The current study borrows with modification, Rizvi and Anam’s (42) use of the Augmented Solow model to determine the effect of health expenditures on economic growth in their study of the quality of health institutions in 20 South, East Asia and Pacific developing countries. We use the Solow-Swan growth model to explore the impact of corruption on the health expenditure-health outcomes nexus in Malawi as follows;


Y=A(t).F(K,L)

Where,


Y=Total Output(Health outcomes:Life Expectancy)

K=Capital stock(Physical and Human Capital)

L=Labour Input(Healthier Population−labour productivity)

A(t)=Level of Technology(representing efficiency)

Grounded in the institutional hypothesis, the present study conceptualizes corruption as a distortionary force that weakens public spending efficiency by diverting resources from productive use, inflating procurement costs, and undermining accountability structures (28–34). Within the Solow–Swan growth model, health expenditure represents investment in human capital (K) that enhances labour productivity (L) and consequently improves health outcomes. However, when corruption is pervasive, the productive return on this investment diminishes, reducing the effective contribution of K and slowing technological progress A(t) (54). In empirical terms, this implies that the relationship between health expenditure and health outcomes is not linear, but conditional on the quality of institutions governing resource allocation. Therefore, to capture the mechanism through which corruption alters the productivity of health expenditure, we introduce an interaction term between health expenditure and corruption in the empirical model. This allows us to directly test whether increased spending improves health outcomes only under conditions of effective governance, and whether corruption significantly weakens or reverses this effect over time.

#### 3.4.2 Model specification

The current study employed bounds cointegration and the Autoregressive Distributed Lag (ARDL) approach to analyse data. The technique was directed by stationarity tests indicating the absence of a unit root only after first difference I(1) in all variables except one. In an intuitive sense, variables that are stationary of order one rule out the option of applying Ordinary Least Squares, which would result in spurious regressions (55, 56). As such, when the stationarity tests indicate variables integrated of I(0) or I(1) or both, the ARDL model is more appropriate (57).

The following econometric specification of the ARDL model shows Life expectancy as the dependent variable.


$$\begin{array}{l} \text{ΔlnLEXP}=\propto_{0}+\sum_{i=1}^{p}\emptyset_{i}\text{ΔlnLEX}P_{t-1}+\sum_{j=0}^{q}\beta_{j}\Delta_{t-j}\\ +\text{γlnLEX}P_{t-1}+\delta X_{t-1}+\varepsilon_{t} \end{array}$$

$\mathrm{LEX}P_{t-1}$ represents the lag of the dependent variable. In ARDL models, the lag of the dependent variable is included as an explanatory variable, hence the name dynamic model (58). X is the vector of independent variables, ∆ is the first difference operator, while p and q denote the lag order of the dependent and independent variables, respectively. In explicit form, with our variables, the specification follows,


$$\begin{array}{l} \text{ΔlnLEXP}=\alpha_{0}+\sum_{i=1}^{p}\alpha_{1i}\text{ΔlnLEX}P_{t-1}+\sum_{J=0}^{q1}\alpha_{2i}\text{ΔlnCR}C_{t-j}\\ +\sum_{j=0}^{q2}\alpha_{3i}\Delta \ln\mathrm{HXP}C_{t-j}+\sum_{j=0}^{q3}\alpha_{4i}(\text{ΔlnHXP}C_{t-j}*\text{ΔlnCR}C_{t-j})\\ +\sum_{j=0}^{q4}\alpha_{4i}\text{ΔlnGDPP}C_{t-j}+\sum_{j=0}^{q5}\alpha_{5i}\text{lnMY}S_{t-j}+\delta_{1}\text{ΔlnLEX}P_{t-1}\\ +\delta_{2}\text{ΔlnCR}C_{t-1}+\delta_{3}(\text{ΔlnHXP}C_{t-1}*\text{ΔlnCR}C_{t-1})\\ +\delta_{4}\text{ΔlnGHE}X_{t-1}+\delta_{5}\Delta \ln\text{GDPP}C_{t-1}+\delta_{6}\text{lnMY}S_{t-1}+\varepsilon_{t} \end{array}$$

In the presence of a long-run relationship among the model variables, the above ARDL model of order (p, q1, q2, q3, q4) is presented using a vector equilibrium or error correction model (ECM), whose coefficients offer an Engle-Granger causality of explanatory variables as shown below,


$$\begin{array}{l} \text{ΔlnLEXP}=\alpha_{0}+\sum_{i=1}^{p}\alpha_{1i}\text{ΔlnLEX}P_{t-1}+\sum_{J=0}^{q1}\alpha_{2i}\text{ΔlnCR}C_{t-j}\\ +\sum_{j=0}^{q2}\alpha_{3i}\Delta \ln\mathrm{HXP}C_{t-j}+\sum_{j=0}^{q3}\alpha_{4i}(\text{ΔlnHXP}C_{t-j}*\text{ΔlnCR}C_{t-j})\\ +\sum_{j=0}^{q4}\alpha_{4i}\text{ΔlnGDPP}C_{t-j}+\sum_{j=0}^{q5}\alpha_{5i}\text{lnMY}S_{t-j}+\mathrm{\theta EC}T_{t-1}+\varepsilon_{t} \end{array}$$

The notation θ represents the model’s speed of adjustment parameter, and the ECT is the error correction term. θ is expected to be negative and statistically significant to confirm a cointegration relationship among the variables. Notably, the ARDL model uses a generalised likelihood estimation approach, so that the lag length is obtained from information such as the Schwarz Bayesian Information (SBI) and the Akaike Information Criteria (AIC).

## 4 Results and discussion

### 4.1 Summary statistics

The summary statistics in Table 1 indicate that life expectancy averaged 52.92047 between 1990 and 2023, with the highest being 64 years and the lowest being 42 years. The current health expenditure per capita is averaging 71 USD, with 24.3 USD as the lowest and $125 USD as the highest expenditure. The corruption index indicates a mean of −0.53, ranging from −1.02 to −0.23, indicating persistent corruption, and with a standard deviation of 0.19, corruption is stable, but severe. The mean years of schooling is averaging 3.93 years which interprets that on average, a Malawian adult aged 25, has only attained 3.9 years of formal education during our study period.

Importantly, pairwise correlation analysis indicate that there is an inverse relationship between corruption and life expectancy. Corruption also negatively affects GDP per capita, health expenditure, and education. Interestingly, other than corruption, all other variables are positively associated with each other. Since correlation does not infer causation, the next section presents results from advance analysis using regression.

**Table 1:** Descriptive and correlation statistics.

	LEXP	CRC	HXPC	GDPPC	MYS
Mean	52.92	−0.537	71.89	118.049	3.9319
Variance	66.16	0.039	881.31	113,858.1	1.3014
Std. dev.	8.133	0.197	29.69	337.43	1.1408
Min.	42.85	−1.02	24.3	671.50	2.2774
Max.	64.12	−0.23	125.8	1,829.5	5.29342
Skewness	0.022	−3.362	0.0482	0.0888	−1.1141
Kurtosis	1.382	2.336	1.8934	1.8427	1.4243
Obs.	34	34	34	34	34
LEXP	1.0000				
CRC	−0.4414	1.0000			
HXPC	0.8010	−0.1635	1.0000		
GDPPC	0.9300	−0.2499	0.7067	1.0000	
MYS	0.9908	−0.4434	0.7464	0.9460	1.0000

Source: Authors’ analysis.

### 4.2 Stationarity test results

One of the most important properties of time series analysis is that the data must be stationary. This property circumvents problems associated with the non-stationarity of time series, such as spurious regression and persistence of shocks. The study used the Augmented Dickey-Fuller (ADF) test for series stationarity, under intercept and trend specifications. The trend component was retained for all variables because the visual plots and statistical significance showed a deterministic trend, except MYS, LEXP, and GDPPC. Importantly, a log-transformation was applied to all variables to ensure interpretability of elasticities and to stabilise variances across variables. Regarding control of corruption, the negative values were shifted by adding a constant equal to |min(CRC)| + 1.5 to allow variable transformation into natural logarithm. This technique preserved the relative distance between data points of the series while converting it to a positive domain without changing the underlying trend, distributional shape, or long-run dynamics.

The ADF test results in Table 2 indicate that we fail to reject the null hypothesis that the series has a unit root (random walk) for life expectancy, control of corruption, health expenditure and gross domestic product per capita, until these variables were first differenced. In contrast, the *p*-value of the mean years of school is significant at 5% level, allowing us to reject the null hypothesis. Thus, one variable is stationary in level, and the rest after first differencing. A mixture of series in *I*(0) and *I*(1) supports the utility of the ARDL model appropriate for our data (21, 36). In addition, the ARDL model is more suited to establishing long-run relationships between variables in small samples than the Johansen alternative methods, which work well with large samples (59).

**Table 2:** Augmented Dickey-Fuller (ADF) test.

Variable	Level			First differencing			
	*t*-statistic	5% CV	Prob	Variable	*t*-statistic	5% CV	Prob.
lnLEXP	−0.303	−2.978	0.9251	ΔlnLEXP	−1.714	−1.701	0.049
lnCRC	−2.525	−2.978	0.1094	ΔlnCRC	−2.087	−1.701	0.023
lnHXPC	−0.805	−2.978	0.8077	ΔlnHXPC	−3.073	−1.701	0.002
lnGDPPC	−0.838	−2.978	0.8077	ΔlnGDPPC	−2.807	−1.701	0.005
lnMYS	−2.748	−2.978	0.0229	…	…	…	…
lnMORT	−1.431	−1.701	0.0818	ΔlnMORT	−2.358	−1.703	0.0129

Source: Authors’ analysis.

### 4.3 Cointegration test results

The Johansen cointegration test cannot be applied since the variables are integrated into a mix of orders zero and one. The Bounds cointegration test was more appropriate, using the Akaike Information Criteria (AIC = −24.65), which yielded a maximum lag order of 3. While all variables are treated as dependent variables in the ARDL model (60), we only tested the bounds on the dependent variables stated in the objective of the study. Table 3 reports the computed *F*-statistic of 11.168 and 4.824, higher than the upper bound at all critical values (1–10%).

**Table 3:** Bounds cointegration test results.

Dep. var	Lag	Matrix list	*F*-stat	Lower bound (5%)	Upper bound (5%)	Outcome
ΔlnLEXP	3	2,3,3,3,3,1	11.168	2.96	4.18	Cointegration
ΔlnMORT	3	2,0,0,0,0,1	4.824	2.62	3.79	Cointegration

The asymptomatic bounds are obtained from Table C2 of case III unrestricted intercept, with no trend for *k* = 3 (Pesaran et al., 2001). Lower bound I(0) = 2.74 and upper bound I(1) = 4.85 at 5% significance level.

Source: Authors’ analysis.

### 4.4 Error correction model estimation results

Given that the results of the cointegration analysis indicate a long-run relationship between life expectancy and the explanatory variables, the study proceeded to estimate the long-run and short-run effects using the ARDL framework to achieve the study’s objective. Table 4 below represents the ARDL (2,3,3,3,3,1) model obtained by normalising life expectancy as a dependent variable of interest. On the other hand, Model 2 of ARDL (2,0,0,0,0,1) lag is used for robustness checks as it is normalised on the under-five mortality rate. The zeroes in the lag order of Model 2 confirms that Malawi achieved reducing the infant mortality rate outlined in Millennium Development Goal III (33, 61). Following Pesaran et al. (69) bounds cointegration test, a maximum of three lags were specified for each dependent variable. Thereafter, the AIC automatically selected these lag combinations as the most appropriate specification within the VARSOC and ARDL routines in STATA. The specification offers the best balance between model fit and parsimony.

**Table 4:** The estimated short and long-run results.

	Model 1 (2,3,3,3,3,1)		Model 2 (2,0,0,0,0,1)	
	Coefficient	Std. err.	Coefficient	Std. err.
Long-run results				
ECT	−1.1373***	0.2129	−0.5741***	0.1098
∆lnCRC	−0.0396**	0.0165	0.0226**	0.0151
∆lnHXPC	0.0178	0.0245	−0.0924***	0.0211
∆lnHXPC* ∆lnCRC	0.3176***	0.0699	−0.1496*	0.0904
∆lnGDPPC	−0.1514***	0.0414	0.09677*	0.0503
lnMYS	0.0104	0.0063	−0.0228*	0.0118
Short-run results				
∆lnLEXP/MORT	0.2912	0.2392	0.6248	0.1445
∆lnCRC	0.0256	0.0170	…	…
∆lnCRC_T-1_	0.0211	0.0129	…	…
∆lnCRC _T-2_	0.0149	0.0109	…	…
∆lnHXPC	−0.0138	0.0187	…	…
∆lnHXPC_T-1_	−0.0178	0.0141	…	…
∆lnHXPC_T-2_	−0.0332**	0.0114	…	…
(∆lnHXPC* ∆lnCRC)	−0.3359***	0.0813	…	…
(∆lnHXPC* ∆lnCRC)_T-1_	−0.3788***	0.0811	…	…
(∆lnHXPC* ∆lnCRC)_T-2_	−0.2109***	0.0531	…	…
∆lnGDPPC	0.1459***	0.0419	…	…
∆lnGDPPC_T-1_	0.1023**	0.0364	…	…
∆lnGDPPC_T-2_	0.0413*	0.0224	…	…
lnMYS	0.5351***	0.0919	−0.2829	0.1105
_cons	−0.0142	0.0109	−0.0079	0.0109

… denotes ‘not applicable’, ***, **, * indicate 1, 5, and 10% significant levels.

Source: Authors’ analysis.

In line with the objectives of the study, both health expenditure and corruption were log-transformed and included in their differenced form to estimate short-run effects within the ARDL framework. Beyond capturing their individual impacts on life expectancy, the model also incorporates an interaction term, expressed as ( Δlnhxppc×Δlncorr) . The inclusion of this interaction term allows us to examine whether variations in corruption intensify or dampen the effect of changes in health expenditure on life expectancy.

While the long-run coefficient of health expenditure positively affected life expectancy, the relationship turns negative with the contingent role of corruption.

Models 1 and 2 yield a coefficient of determination (*R*^2^) of 0.9223 and 0.8316, which means that the included independent variables explain about 92.23 and 82.16%of the variations in life expectancy in Malawi. The remaining variation are captured by the error term and can be attributed to other factors not included in the model. Importantly, postestimation tests were carried out for the estimated ARDL models, and results show that the models do not suffer from heteroskedasticity (Breusch-Pagan test) or omitted variables (Ramsey RSET). Serial correlation (Durbin-Watson) and residuals are normally distributed (Skewness-kurtosis and Shapiro–Wilk tests), as shown in Table 5.

**Table 5:** Postestimation checks.

Diagnostic	Null hypothesis	Model 1		Model 2	
		Statistic	*p*-value	Statistic	*p*-value
Breusch-Pagan	Constant variance	1.14	0.2851	0.32	0.5709
Ramsey RESET	No omitted variables	0.64	0.5941	0.26	0.8505
Durbin-Watson	No serial correlation	1.8526	…	1.8242	…
Skewness-Kurtosis	Normally distributed	0.04	0.9806	2.75	0.2534
Shapiro–Wilk	Normally distributed	0.98837	0.9726	−0.439	0.6697

**Figure 1:**
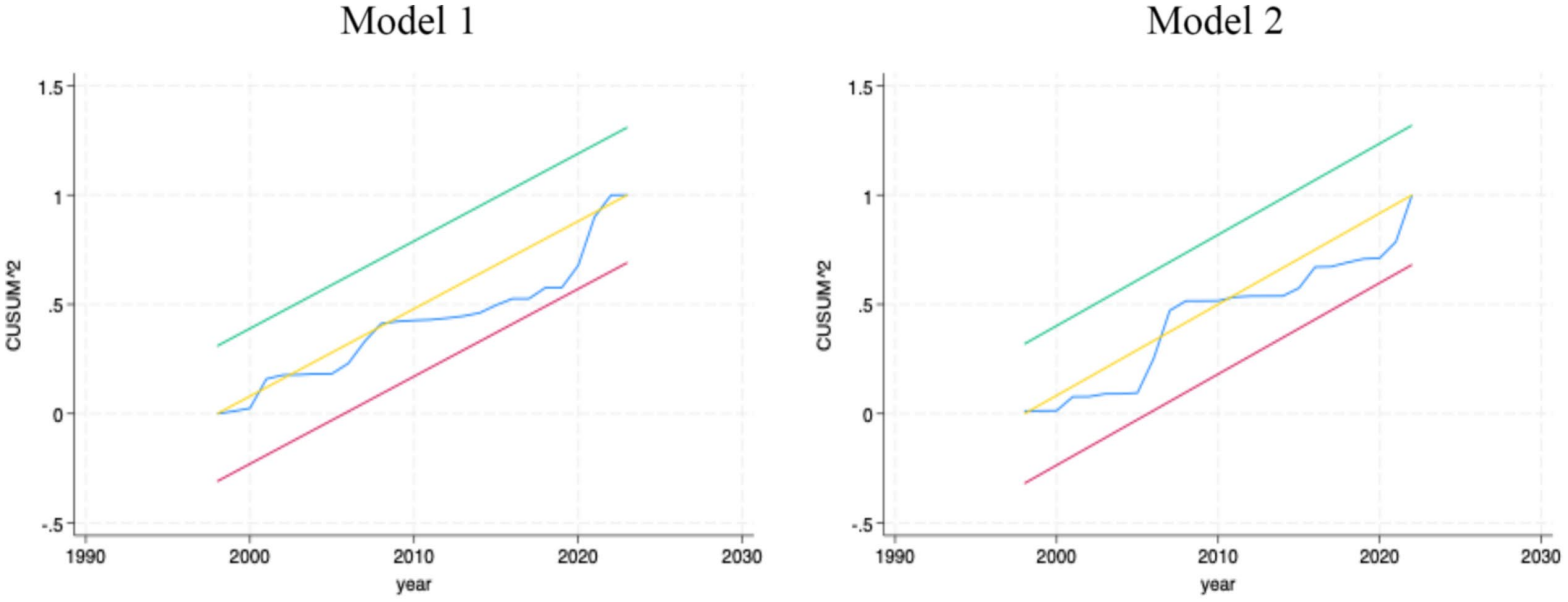
CUSUM-of-Squares (CUSUMSQ) for model stability.

Regarding parameter stability, we computed the cumulative sums (CUSUM9) of the recursive residuals and their squares with a 95% confidence band. The results in Figure 1 show that the series are desirably stable, lying within the upper and lower bounds for the entire study period. Thus, we fail to reject the null hypothesis of the absence of structural breaks in the parameters over the sample period (62). In addition, the error correction term (ECT) coefficients for models 1 and 2, of −1.1373 and −0.5741, are statistically significant with the right sign. These coefficients confirm the results of the bounds cointegration test that there is a long-run relationship among the variables in all our models. Importantly, the result provides evidence of a rapid speed of adjustment back to the equilibrium in the event of shocks or sudden changes in any of the variables.

#### 4.4.1 Model estimation results

Results in Table 5 indicate that corruption does not have a statistically significant effect on life expectancy in the short run. However, in the long run, the estimated coefficient for corruption is negative and statistically significant. Specifically, a 1 percent increase in corruption is associated with a 0.04 percent decrease in life expectancy, holding other factors constant. This elasticity-based interpretation implies that persistent increases in corruption erode institutional efficiency and weaken healthcare delivery systems, which reduces the longevity of the country’s population. A study by Achim et al. (27) in 54 high-income and 131 low-income countries found similar findings that corruption reduces life expectancy and exacerbates under-five and maternal mortality rates. Similarly, Naher et al. (45) found that corruption hinders the delivery of quality healthcare services, leading to poor health outcomes.

Regarding the temporal effects of corruption on life expectancy, we suggest that the deterioration of health outcome from corruption is a gradual process. In the immediate time, the immediate impact of corruption may be cushioned by prevalent institutional structures and other supporting health programmes. As time goes, however, the perpetuation of corrupt practices weakens institutional capacity, distorts resource allocation, and erodes the quality of healthcare services, leading to deterioration in overall population health and longevity. The lagged nature of corruption’s influence on health outcomes aligns with existing empirical findings that poor governance negatively influences health outcomes through long-run institutional decay (63).

The estimated short-run coefficients of GDP per capita are positive and statistically significant at all lags. In the current year, for example, a 1 percent increase in GDP per capita will result in a 0.146 percent increase in life expectancy in Malawi. Contrary to the *a priori* expectation, the long-run coefficient of GDP per capita has a negative and significant relationship with life expectancy. That is, a 1 percent increase in GDP per capita is associated with a 0.1514 percent decrease in life expectancy, at ceteris paribus. This counterintuitive finding may indicate that while short-term economic boosts can improve healthcare access or affordability, long-term growth without equitable distribution or investment in social services fails to sustain these gains. In particular, Malawi has issued many policy reforms aimed at opening the economy to international trade focusing on value addition over raw-material exports. Questions have been raised about the failure to balance investment in capital-intensive or service industries with primary and secondary industries may lead to the failure of building trickle-down effects such as employment (64). Over time, such economic boosts increase unfair distribution of income and limits investment in social sectors such as health and education. Further, the quest for growth in extractive and agricultural dependent countries may result in environmental damage, food shortage, and public health issues that counteracts the benefits of rising income (65). The relationship between growth and health outcomes, therefore, is complex as it is highly contingent on other factors such as the quality, inclusiveness, and sustainability and not solely its expansion (66).

On the other hand, the long-run coefficient of mean years of schooling is positively associated with life expectancy in both periods. For instance, a 1 percent improvement in education standard in Malawi will result in a 0.5351 percent increase in life expectancy in the short run. Similar findings, showing a positive association between education and life expectancy or related health outcomes, are revealed in other studies as well (33, 43). These studies incorporate education as a determinant in models assessing health outcomes, aligning with the positive long-term and short-run effects of mean years of schooling on life expectancy in Malawi.

While the long-run coefficient of health expenditure positively affected life expectancy, the relationship turns negative with the contingent role of corruption. The long-run results are such that a 1 percent increase in the interaction of health expenditure and corruption results in a 0.3176 percent decrease in life expectancy. Notably, the interaction of health expenditure and corruption depicts negative effects on life expectancy at all lags in the short run. This is not far-fetched considering that studies confirm that life expectancy increases when there are better measures for curbing corruption in a country (31, 32). Many various studies have empirically ascertained that corruption negatively affects health outcomes such as life expectancy and mortality rates, and can reduce the effectiveness of health spending (27–29, 31, 32, 45). These studies align with the observed contingent negative effect of corruption on the relationship between health expenditure and life expectancy, particularly in low-income or developing contexts like those in sub-Saharan Africa.

#### 4.4.2 Robustness checks

For robustness checks, we changed the dependent variable to the under-five mortality rate to measure health outcomes. Model 2 of ARDL (2,0,0,0,0,1) lag order showed that corruption increases mortality rate, confirming results of the first model that corruption is an evil cancer that erodes health outcomes. The short-and long-run coefficient of education is negative and statistically significant, validating that an improvement in education is crucial at reducing under-five mortality rate in Malawi. This model, which includes the same variables as the first model, explains 83.16% of the variations in the under-five mortality rate. However, lag order of zero for short-run coefficients of most variables when the regression is normalised on under-five mortality validates our justification of the use of life expectancy (25, 43, 67). Most countries have seen a significant decrease in infant mortality due to improvements in healthcare, sanitation, vaccines, and living standards. Nevertheless, while Asian countries are catching up to Western Europe and North America, closing the life expectancy gap remains a far-fetched dream for most African countries (68). Corruption as a critical cause of poor institutional quality increases under-five mortality rates, while health expenditure and education improvements reduce them (23, 27, 28, 30, 31).

While most of the supporting literature is based on cross-sectional or panel frameworks, the present time-series ARDL approach captures dynamic within-country adjustments over time rather than cross-country averages. The alignment of our results with previous studies therefore reflects consistency in the direction of association between health expenditure, corruption, and health outcomes, even though methodological designs differ.

## 5 Conclusion

This study investigated (1) the impact of health expenditure on health outcomes and (2) the moderating role of corruption in that relationship in Malawi. The research adopted the institutional hypothesis, which posits that the fundamental determinants of economic performance and development are the quality and structure of its institutions. We placed corruption benchmarks as a breakdown in institutional accountability, transparency, and governance, which limits public spending potential to achieve the intended development goals. In the augmented Solow Swan growth model, we hypothesised that health expenditure is an investment in human capital (K), which directly enhances labour (L) by improving productivity. In addition, we described corruption as a source of inefficiency, which reduces the productivity of government investment in health (K) and the rate of technological progress (A(t)). That is, corruption tends to undermine the importance of government investment in health by diverting funds meant for health infrastructure and services, fostering inefficiencies in health goods and service delivery.

The study adopted a time series analysis for data from 1990 to 2023, obtained from the World Bank’s World Development and Governance Indicators, and analysed using STATA 19. The study specified the model such that life expectancy was the outcome variable as a health outcome, with health expenditure and corruption as explanatory variables of interest, while controlling for education and national output. The under-five mortality rate was used as a dependent variable for robustness checks. Importantly, all variables transformed into logs were stationary after first differencing, except mean years of schooling, which was stationary in level, thereby justifying our use of the bounds cointegration test. After confirming a long-run relationship, an Engle-Granger causality (ECM) ARDL model was used to analyse our dataset.

Our findings are such that corruption does not affect life expectancy in the short run. The long-term effects are evident, where a one-unit increase in corruption reduces life expectancy by 0.05 percentage points, holding other factors constant. We also found that while health expenditure increases life expectancy in the long run, the relationship turns negative with the contingent effect of corruption, which worsens health outcomes by 0.3176 percentage points. This negative effect of the interaction of health expenditure and corruption is evident at all lags in the short run. These results confirm both our conceptual institutional hypothesis and empirical framework of the Solow-swan growth model.

In addition, we also found that GDP per capita positively affect life expectancy in the short run, at all lags. However, counterintuitive results were found in the long run. We attributed the phenomena of growth without equitable distribution or investment in social services that fail to sustain the gains and spillover effects to the general population. Coupled with income inequality, such growth does not translate into improved income, affordability and accessibility of healthcare services for the general population. Importantly, mean years of schooling showed expected results for both models, normalised on life expectancy and under-five mortality. The results confirm that the more years adult persons spend in formal education, the more likely it is that a country will experience better health outcomes.

The results reveal the need for multifaceted interventions to improve health outcomes in Malawi. Firstly, this paper recommends prioritising anti-corruption measures, including stricter audits, transparent procurement processes, and accountability mechanisms for public health funds. Moreover, this study’s results suggest that health expenditure should be tied to measurable outcomes to ensure efficiency and reduce leakage. Thirdly, the paper recommends leveraging mediating factors, such as institutional quality and education, which can amplify the impact of health investments. Finally, addressing inequality and ensuring economic growth benefits the broader population are essential for long-term health improvements.

While the ARDL model provides valuable insights, it has limitations. The control of corruption indicator employed is perception-based and is likely to contain reporting bias or measurement error, which may affect the precision of governance estimates. The dependence on yearly time-series data (*n* = 34) also restricts the granularity of institutional shocks and short-term dynamics. In addition, the ARDL approach yields short-run and long-run associations rather than causal effects, such that’s its findings are interpreted as conditional relationships in tandem with theory and not definitive causal estimates. Our use of lag structures and an error correction mechanism helps to reduce issues of reverse causality. We therefore urge future researchers to incorporate alternative governance measures, higher-frequency datasets, or structural break methods to validate the robustness of the moderating effect of corruption. Comparative studies using panel or multi-country designs may also help establish external validity beyond the Malawian context.

## Data Availability

The original contributions presented in the study are included in the article/supplementary material, further inquiries can be directed to the corresponding author.
